# P021 Case report: The Skeletal Consequences of the Primary Hyperoxaluria

**DOI:** 10.1093/rap/rkag113.051

**Published:** 2026-11-03

**Authors:** Qadija Elmi, Mohamed Rizwan Rajak

**Affiliations:** Croydon University Hospital, London, United Kingdom; Croydon University Hospital, London, United Kingdom

## Abstract

**Introduction:**

A 24 year old patient presented with severe wrist pain and swelling with a background of Type 1 Primary hyperoxaluria. She was subsequently treated for this arthritic manifestation of her metabolic condition. On subsequent review of previous imaging, it was noted that the patient has osteodysrophy likely secondary to skeletal oxalosis. This case report will focus on the pathogenesis of renal osteodystrophy secondary to primary hyperoxaluria.

**Case description:**

Type 1 Primary Hyperoxaluria is an autosomal recessive condition leading to deficiency of the enzyme alanine glyoxylate aminotransferase (AGT) secondary to the inheritance of the pathogenic variant of the AGXT gene. (1) The enzyme AGT normally converts glyoxalate to glycine. Due to the lack of AGT in type 1 primary hyperoxaluria, the glyoxalate instead is converted to endogenous oxalate which leads to accumulation of oxalate. (1)

Oxalate is usually renally excreted, however due to the elevated levels of urinary oxalate, this leads to formation of calcium oxalate crystals which then accumulate leading to renal failure and deposition of calcium oxalate elsewhere including skeletal deposits. (1)

In this case, the patient had initially presented with recurrent calcium oxalate renal stones requiring intervention with percutaneous nephrolithotomy, after multiple presentations and interventions as a paediatric patient; she was referred for genetic investigations at age 18 which led to diagnosis of her inherited metabolic condition. She subsequently developed end stage renal failure requiring dialysis and has since been found to have dermatological, ophthalmological and skeletal complications.

Her early XR and CT scans do not show renal osteodystrophy, however her recent scans over the last year show hyperostosis. In renal osteodystrophy, there is both the primary method of damage e.g. skeletal deposition of calcium oxalate crystals, as well as a secondary cause which is secondary hyperparathyroidism due to renal failure. (2)

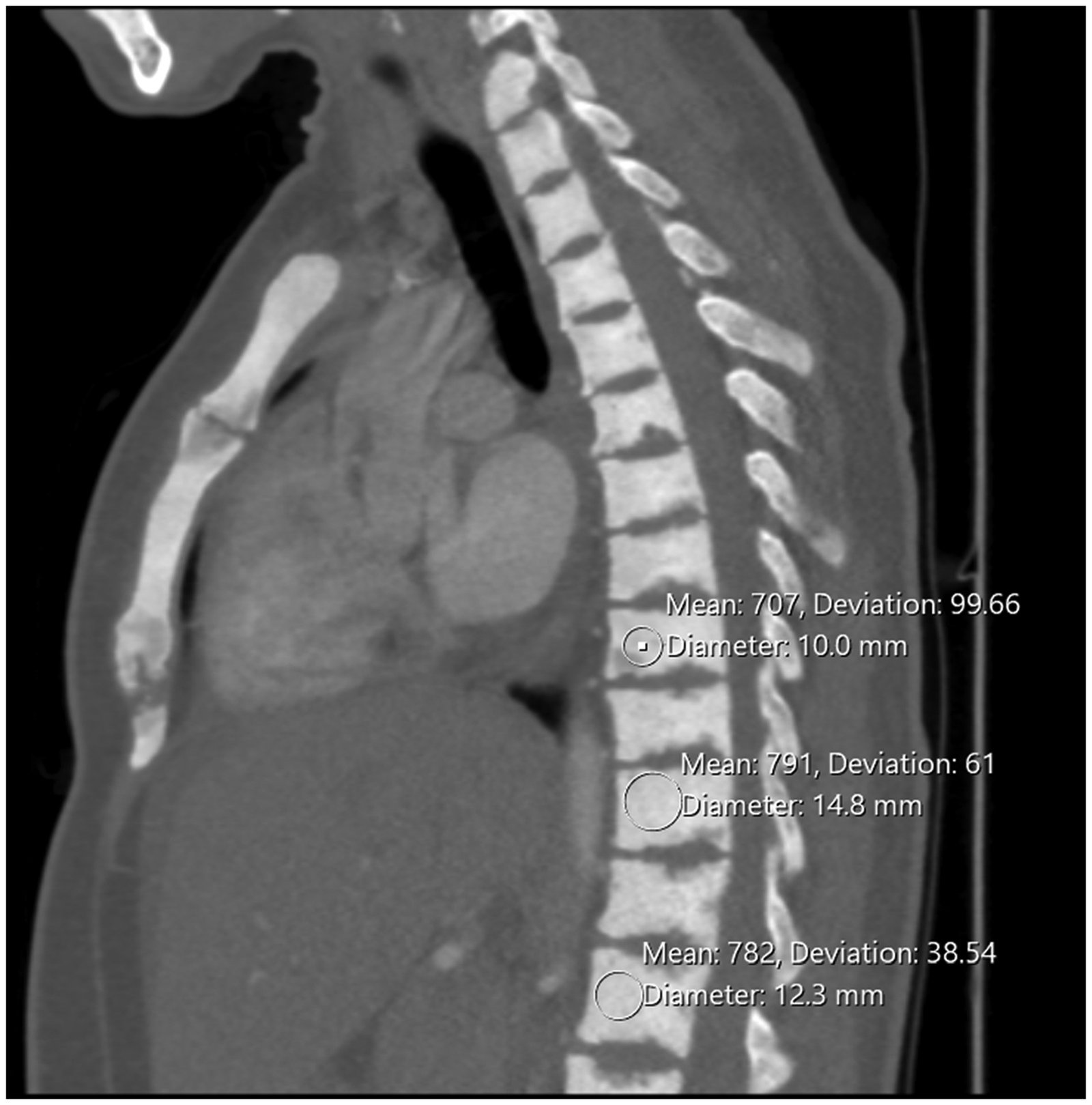

**P021 Figure 1**

**Reference:**

1. Fargue S, Acquaviva Bourdain C. Primary hyperoxaluria type 1: pathophysiology and genetics. Clin Kidney J. 2022 May 17;15(Suppl 1):i4-i8. doi: 10.1093/ckj/sfab217. PMID: 35592619; PMCID: PMC9113437.

2. Benhamou CL, Pierre D, Geslin N, Viala JF, Maitre F, Chavassieux P, Edouard C, Meunier PJ. Primary bone oxalosis: the roles of oxalate deposits and renal osteodystrophy. Bone. 1987;8(2):59-64. doi: 10.1016/8756-3282(87)90071-8. PMID: 3593609.

**Discussion:**

Skeletal oxalosis triggers a “granulamatous macrophagic reaction” leading to bone resorption and increased osteoclast activity (2). This is seen as both lucency due to bone resorption and damage to microarchitecture as well as hyperostosis due to the oxalosis.(3)

Radiologically, this often manifests as “dense and radiolucent metaphyseal bands and verterbral osteocondensations” (3) which are a result of the skeletal oxalosis as well as renal osteodystrophy due to hyperparathyroidism.

This can lead to high risk of low impact fracture and due to the inherited nature of this metabolic condition, this is present in young individuals for whom often skeletal consequences are not considered. This particular patient on review of her clinic visits had not had this noted within her records. Due to the severe renal and cardiac manifestations of her condition, the skeletal oxalosis was not highlighted.

Overall the importance of managing these patients and monitoring them for potential hyperostosis and increased risk of fracture is crucial.

**References:**

1. Benhamou CL, Pierre D, Geslin N, Viala JF, Maitre F, Chavassieux P, Edouard C, Meunier PJ. Primary bone oxalosis: the roles of oxalate deposits and renal osteodystrophy. Bone. 1987;8(2):59-64. doi: 10.1016/8756-3282(87)90071-8. PMID: 3593609

2. El Hage S, Ghanem I, Baradhi A, et al. Skeletal features of primary hyperoxaluria type 1, revisited. *Journal of Children’s Orthopaedics*. 2008;2(3):205-210. doi:10.1007/s11832-008-0082-4

**Key learning points:**

1. To consider skeletal complications in patients with inherited metabolic conditions

2. To have routine skeletal review of patients with known inherited conditions with risk of renal osteodystrophy

3. Consider preventative measures for patients with above conditions

